# LncRNAs as Theragnostic Biomarkers for Predicting Radioresistance in Cancer: A Systematic Review and Meta-Analysis

**DOI:** 10.3389/fonc.2022.767750

**Published:** 2022-05-25

**Authors:** Ping Lin, Wenmin Xing, Qian Ren, Qin Wang, Jing Yan, Genxiang Mao

**Affiliations:** ^1^ Department of Geriatrics, The Third People’s Hospital of Hangzhou, Hangzhou, China; ^2^ Zhejiang Provincial Key Laboratory of Geriatrics, Department of Geriatrics, Zhejiang Hospital, Hangzhou, China

**Keywords:** long non-coding RNAs, radioresistance, theragnostic biomarkers, cancer, meta-analysis

## Abstract

**Background:**

Radioresistance is the major obstacle after cancer radiotherapy. The dysregulation of long non-coding RNAs (lncRNAs) was closely related the radioresistance response. This meta-analysis was aimed to interpret the relationship between lncRNAs and radiotherapy responses in different cancers.

**Method:**

The studies were selected from databases including PubMed, ISI Web of Science, Embase, Google Scholar, PMC, and CNKI (China National Knowledge Infrastructure). The publication time was limited to before March 20, 2021. The hazard ratios (HRs) and 95% confidence interval were calculated with random-effects models. Subgroup analyses, sensitivity analyses, and publication bias were also conducted.

**Result:**

Twenty-seven lncRNAs in 14 cancer types were investigated, in which 23 lncRNAs were upregulated and four lncRNAs were downregulated. Dysregulation of these lncRNAs were found to be related to radioresistance response. The pooled HR and 95% confidence interval for the combined up-regulated lncRNAs was 1.73 (95% CI=1.50-2.00; P< 0.01) and down-regulated lncRNAs was 2.09 (95% CI= 1.60-2.72; P< 0.01). The HR values of the subgroup analysis for glioma (HR= 2.22, 95% CI= 1.79-2.74; p< 0.01), non-small cell lung cancer (HR=1.48, 95% CI=1.18-1.85; P<0.01), nasopharyngeal carcinoma (HR=4.26; 95% CI= 1.58-11.46; P< 0.01), and breast cancer (HR=1.29; 95% CI= 1.08-1.54; P< 0.01) were obtained. Moreover, the expression of lncRNAs was significantly related to overall survival of patients no matter if the sample size was >50 or not. In addition, the HR values of the subgroup analysis for lncRNA H19 (HR=2.68; 95% CI= 1.92-3.74; P <0.01), lncRNA FAM201A (HR=2.15; 95% CI= 1.15-3.99; P <0.01), and lncRNA HOTAIR (HR=1.22; 95% CI= 0.98-1.54; P =0.08) were also obtained.

**Conclusion:**

LncRNAs can induce cancer radioresistance by regulating cell death-related signaling pathways. Results indicated that lncRNAs, especially lncRNA H19, FAM201A, and HOTAIR, could be considered as a predictive theragnostic biomarker to evaluate radiotherapy response.

## Introduction

Cancer is one of the dominant factors causing global death, and deaths caused by cancer have far exceeded those caused by infectious diseases such as human immunodeficiency virus (HIV) and tuberculosis (TB) (1). GLOBOCAN estimated that 19.3 million new cases of cancer and almost 10.0 million deaths from cancer occurred in 2020 (2). Worldwide, the cancer burden is expected to be 28.4 million cases in 2040, which is a 47% rise from 2020. Limited access to timely diagnosis and effective treatment led to disparities in cancer survival between different locations. Advances in surgery, chemotherapy, radiotherapy, as well as other newer targeted therapies, had significantly reduced morbidity and improved the overall survival of cancer patients over the past decades (3). Among these treatments, about one-half of cancer patients with persistent or recurrent tumors receive radiotherapy (RT) (4, 5). However, radioresistance, a major hurdle for the clinical cancer treatment, widely induced deterioration of cancer including invasion, metastasis, poor prognosis, and overall survival of cancer patients (6, 7). Radioresistance is involved in multiple biological changes, for example, evasion of apoptosis, altered DNA damage response, and enhanced DNA repair (8). As a result, patients with radioresistant characteristics always require higher doses of irradiation to obtain effective treatment, but this treatment manner would lead to more serious side effects (9). Therefore, it is essential to explore novel biological markers that lead to radioresistance or radiosensitivity to benefit the treatment of radioresistant cancer patients and improve the outcome of radiotherapy.

Recently, it has been demonstrated that the dysregulation of long non-coding RNAs (lncRNAs) after ionizing radiation (IR) was different between radioresistant and radiosensitive patients (10–14). LncRNAs, a series of transcripts of more than 200 nucleotides, are generally defined as no or limited protein-coding potential (15). Numerous studies have indicated that lncRNAs are involved in gene expression regulating, transcription modulation, post-transcription modulation, as well as epigenetic modification of biological process (16, 17). Moreover, lncRNAs also play a vital role in multiple signal transduction pathways in cancer progression and metastasis, including miRNA silencing, DNA damage, cell cycle control, and hormone-driven disease states (18). Currently, a series of functional lncRNAs have been identified as key factors to induce radioresistance response by regulating the expression of their target genes at transcriptional or post-transcriptional levels (19–21). For instance, lncRNACCAT2 and lncRNARpph1 as strategic components augmenting the radiation therapy in esophageal squamous cell carcinoma (22, 23). Besides, lncRNA-p21 was indicated to promote the radiotherapy sensitivity of gastric cancer (24). Moreover, many lncRNAs such as lncRNATP73-AS1 (25), lncRNAFAM201A (26), lncRNA01600 (27), lncRNANCK1-AS1 (28), lncRNAMAGI2-AS3 (29), and lncRNAAGAP2-AS1 (30) have also been proven to participate in radioresistance response during cancer treatment. In addition, a recent study also identified that lncRNA FAM133B-2 showed radiosensitivity response in nasopharyngeal cancer cells by targeting miR-34a-5p/CDK6 axis (31).

However, these previous studies only focused on one single lncRNA based on small sample size or one study center. The association between lncRNAs and cancer radioresistance is still poorly understood. Therefore, more functional lncRNAs related to the radioresistance or radiosensitivity response need to be explored based on multiple centers and big sample size. Meta-analysis pooled large amount of available data and provided more precise estimates. Therefore, this study was performed to provide novel insights into lncRNAs associated with radioresistance and radiosensitivity of various cancers by a systematic review and meta-analysis. Studies were selected based on the inclusion and exclusion criteria. The extracted HRs and the corresponding 95% CIs were calculated followed by sensitivity analyses, publication bias assessment, as well as subgroup analysis.

## Materials and Methods

The present meta-analysis was guided by the Preferred Reporting Items for Systematic Reviews and Meta-analysis (PRISMA) guidelines (32).

### Search Strategy

The topic related literature was searched by two authors independently through PubMed, PMC, ISI Web of Science, Embase, Google Scholar, Cochrane Library, the Chinese National Knowledge Infrastructure (CNKI), and Chinese Biomedical Literature Database (CBM). The studies were limited to English or Chinese language which published before June 30, 2021. The terms “long non-coding RNA,” “lncRNAs,” “radioresistance,” “radiosensitivity,” “cancer,” “tumors,” “neoplasm,” and “overall survival” were used to search the related studies. Articles in the references listed in the studies were also searched to avoid missing the relevant studies.

### Inclusion and Exclusion Criteria

Inclusion and exclusion criteria were chosen as the following. Retrieved studies were included if they met the following characteristics: 1) human individuals; 2) the diagnosis of cancer disease was clinically confirmed according to the disease guideline; 3) the expression of lncRNAs and its related overall survival were evaluated; 4) lncRNAs were involved in radioresistance or radiosensitivity in cancer therapy; 5) case group size, control group size, HR value, and its related 95%CIs were provided; 6) the publication language was limited to Chinese or English.

Studies were excluded if they met the following characteristics: 1) cell line or animal studies, case reports, comments, abstract, or review articles; 2) not related to radioresistance or radiosensitivity and cancer; 3) repeated studies; 4) insufficient data to calculate the HR value; 5) studies not related to the topics.

### Data Extraction and Quality Assessment

Two authors retrieved the following data independently from the included studies: first author, publication year, country, sample characteristic, sample source, type of cancer, involved lncRNAs, detection method, related therapy, the case and control size, and the HR value and its 95CIs. If the included studies didn’t provide detailed data, the HR value and its 95CIs would be calculated based on the overall survival curves. If there was any inconsistency, the third researcher checked and resolved.

For further assessment of the quality of the included studies, two of the authors independently evaluated studies using the Newcastle-Ottawa Scale (NOS) (33). Studies scoring less than 6 were excluded in the present meta-analysis. Disagreements were resolved by discussion.

### Statistical Analysis

The hazard ratios (HRs) with 95% confidence intervals (CIs) of lncRNAs and patients in included studies were extracted, and then the HRs and 95% CI as the effect magnitude were calculated as the amount of the combined effect. Subgroup analysis was conducted according to cancers difference, case sample size >50 or not, and the different expression trend of lncRNAs. The heterogeneity across studies was assessed using the *I^2^* statistic. When *I^2^* value was 25%, 50%, and 75%, it was low, moderate, and high degrees of heterogeneity, respectively. The pooled effect was conducted by the random-effects model when *I* ^2^ value was > 75%. Otherwise, a fixed-effects model was used. The Begg and Egger’s tests were used to assess potential publication bias. All statistical analyses were conducted by Stata software version 12.0 (Stata Corp LP, TX, USA). The statistical significance was considered as a P-value< 0.05.

## Results

### Characteristics and Quality of the Included Studies and Individuals

**Figure 1:**
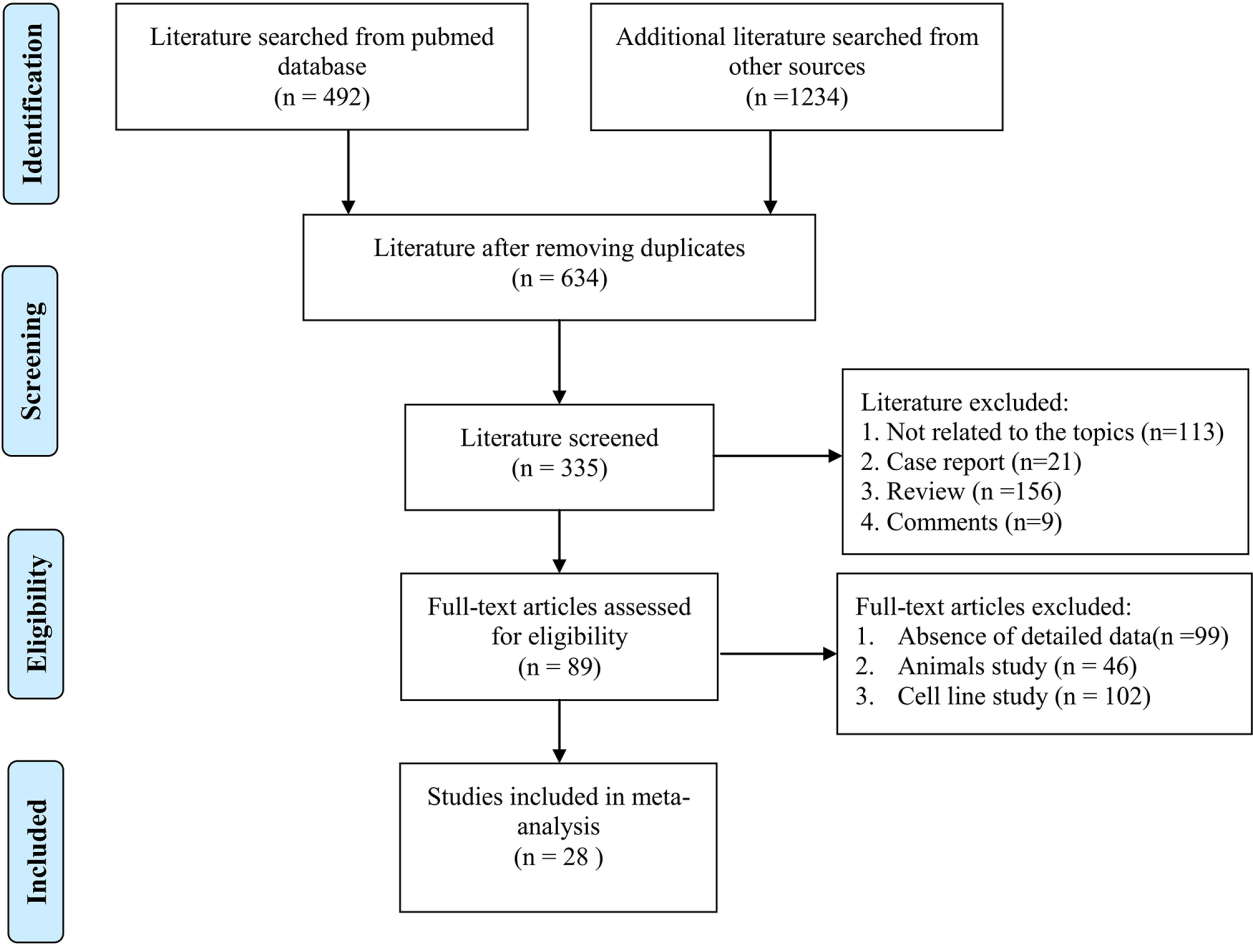
The diagram flowchart of studies for selecting process.

**Figure 1** shows the flowchart of study selection and exclusion. Three hundred eleven studies were initially searched from PubMed (n = 492), and other databases (n = 1234). After removing duplicated studies, 634 articles were considered relevant to the topic. After full-text screening according to inclusion and exclusion criteria, some records were removed because they were case report, review, or comments. Finally, a total of 28 studies with lncRNAs expression involved in radiosensitivity or radioresistance during cancer therapy were included, which contained a total of 4986 cancer patients.

The characteristics of the studies and individuals included are presented in **Table 1**. The results of NOS quality score of the eligible studies ranged from 6 to 9, as also illustrated in **Table 1**. And then, the eligible articles were further reviewed and data extracted. The included studies in the current systematic review and meta-analysis were published between 2015 and 2020 and conducted in Canada and China. The sources of the malignant tumor included glioma (35, 36, 38, 41, 43), neck squamous carcinoma (HNSCC) (57), esophageal cancer (EC) (22), breast cancer (34, 42, 46, 47), colorectal cancer (39, 58), non-small cell lung cancer (NSCLC) (40, 45, 50–52), ovarian cancer (OC) (48), cardiac carcinoma (49), thyroid carcinoma (44), prostate cancer (54), and nasopharyngeal carcinoma (NPC) (37, 56). Sample sizes included in these studies varied from 31 to 955 between the studies. The frozen or formalin-fixed tissue samples were used in these eligible studies, and lncRNAs were detected by qPCR or qRT-PCR assay.

**Table 1:** Basic characteristics of the included studies.

Authors	year	Country	Type of cancer	Clinical Stages	lncRNA	Therapy effect	No. of samples (radiosensitive/radioresistant)	Types of samples	lncRNAdysregulation	NOSscore
**Li et al.** (34)	**2021**	**China**	**breast cancer**	**I-II/35, III- IV/15**	**FGD5-AS1**	**Radioresistance**	**25/25**	**tissue**	**upregulated**	**8**
**Kuang and Bing** (35)	**2021**	**China**	**glioma**	**NM**	**H19**	**Radioresistance**	**202/202**	**tissue**	**upregulated**	**9**
**Li et al.** (36)	**2021**	**China**	**glioma**	**NM**	**DRAIC**	**Radioresistance**	**68/69** **154/154**	**tissue**	**downregulated**	**6**
**Guo et al.** (37)	**2021**	**China**	**NPC**	**NM**	**LINC00312**	**Radioresistance**	**40/41**	**tissue**	**upregulated**	**7**
**Lin et al.** (38)	**2020**	**China**	**glioma**	**II/52,III/115**	**AC106786.1** **LINC02237** **LINC01447**	**Radioresistance**	**124/43**	**tissue**	**upregulated**	**5**
**Liu et al.** (39)	**2020**	**China**	**colorectal cancer**	**NM**	**LINC00630**	**Radioresistance**	**25/25**	**tissue**	**upregulated**	**6**
**Qin et al.** (40)	**2020**	**China**	**NSCLC**	**NM**	**LINC00473**	**Radioresistance**	**38/34**	**tissue**	**upregulated**	**6**
**Tang etal.** (41)	**2021**	**China**	**glioma**	**NM**	**LINC01057**	**Radioresistance**	**81/41**	**tissue**	**upregulated**	**7**
**Li et al.** (34)	**2020**	**China**	**HNSCC**	**NM**	**LINC00520**	**Radioresistance**	**130/389**	**tissue**	**upregulated**	**9**
**Wang et al.** (23)	**2020**	**China**	**EC**	**NM**	**CCAT2**	**Radioresistance**	**34/26**	**tissue**	**upregulated**	**6**
**Zhang et al.** (42)	**2020**	**China**	**breast cancer**	**NM**	**HOTAIR**	**Radioresistance**	**428/427**	**tissue**	**upregulated**	**9**
**Tang et al.** (43)	**2020**	**China**	**glioma**	**I−II/105, III- IV/72**	**TPTEP1**	**Radioresistance**	**96/81**	**tissue**	**downregulated**	**7**
**Liu et al.** (44)	**2020**	**China**	**colorectal cancer**	**NM**	**HOTAIR**	**Radioresistance**	**35/36**	**tissue**	**upregulated**	**6**
**Li et al.** (22)	**2020**	**China**	**EC**	**I-II/56, III- IV/27**	**Rpph1**	**Radioresistance**	**41/42**	**tissue**	**upregulated**	**6**
**Liu et al.** (45)	**2019**	**China**	**NSCLC**	**II/15, III/54**	**FAM201A**	**Radioresistance**	**37/32**	**tissue**	**upregulated**	**7**
**Wang et al.** (46)	**2019**	**China**	**breast cancer**	**NM**	**LINC02582**	**Radioresistance**	**71/65**	**tissue**	**upregulated**	**7**
**Liu et al.** (47)	**2019**	**China**	**breast cancer**	**NM**	**LINC00511**	**Radioresistance**	**49/49**	**tissue**	**upregulated**	**7**
**Dou et al.** (48)	**2019**	**China**	**OC**	**I-II/32, III-IV/48**	**FAM83H-AS1**	**Radioresistance**	**38/42**	**tissue**	**upregulated**	**7**
**Jia et al.** (49)	**2019**	**China**	**cardiac carcinoma**	**I-II/185,III-IV/99**	**H19**	**Radioresistance**	**191/93**	**tissue**	**upregulated**	**8**
**Zhang et al.** (50)	**2018**	**China**	**NSCLC**	**I-II/29,III/35**	**CYTOR**	**radiosensitivity**	**32/32**	**tissue**	**upregulated**	**6**
**Yang et al.** (51)	**2019**	**China**	**NSCLC**	**II/53,III/35**	**LINC00483**	**Radioresistance**	**127/375**	**tissue**	**upregulated**	**8**
**Yang et al.** (52)	**2018**	**China**	**laryngeal cancer**	**I-II/29, III- IV/36**	**NKILA**	**Radioresistance**	**32/33**	**tissue**	**downregulated**	**6**
**Liu et al.** (44)	**2018**	**China**	**thyroid carcinoma**	**NM**	**MEG3**	**Radioresistance**	**24/24**	**tissue**	**downregulated**	**6**
**Chen et al.** (26)	**2018**	**China**	**Squamous Cell Cancer**	**II/2,III/14,IV/25**	**FAM201A**	**Radioresistance**	**12/22**	**tissue**	**upregulated**	**6**
**Zhang et al.** (53)	**2018**	**China**	**LAD**	**I–II/36, III/30**	**CRNDE**	**Radioresistance**	**36/30**	**tissue**	**upregulated**	**7**
**Ghiam et al.** (54)	**2017**	**Canada**	**prostate cancer**	**NM**	**UCA1**	**Radioresistance**	**209**	**tissue**	**upregulated**	**8**
**Wu et al.** (55)	**2017**	**China**	**NSCLC**	**NM**	**PVT1**	**Radioresistance**	**15/16**	**tissue**	**upregulated**	**6**
**Jin et al.** (56)	**2015**	**China**	**NPC**	**I-II/70, III- IV/61**	**MALAT1**	**Radioresistance**	**26/18**	**tissue**	**upregulated**	**6**

NSCLC, non-small cell lung cancer; NPC, nasopharyngeal carcinoma; HNSCC,neck squamous carcinoma; EC, esophageal cancer; OC, ovarian cancer; LAD, lung adenocarcinoma; NM, Not Mentioned.

### Systematic Review

In total, 27 lncRNAs in 14 cancer types were studied in our systematic review and meta-analysis. Twenty-three lncRNAs were upregulated and four were downregulated compared with healthy individuals. Higher expression of lncRNA01057 in glioma, lncRNA00520 in HNSCC, lncRNA CCAT2 in EC, lncRNA HOTAIR in breast cancer and colorectal cancer, lncRNA Rpph1 in EC, lncRNA FAM201A in NSCLC, lncRNA LINC02582 in breast cancer, lncRNA 00511 in breast cancer, lncRNA FAM83H-AS1 in OC, lncRNA H19 in cardiac carcinoma, lncRNA CYTOR in NSCLC, lncRNA 00483 in NSCLC, lncRNA UCA1 in prostate cancer, lncRNA PVT1 in NSCLC, lncRNA MALAT1 in NPC were associated with radioresistance. While lower expression of lncRNA TPTEP1, DRAIC in glioma, lncRNA NKILA in laryngeal cancer, and lncRNA MEG3 in thyroid carcinoma were correlated to radioresistance.

**Figure 2:**
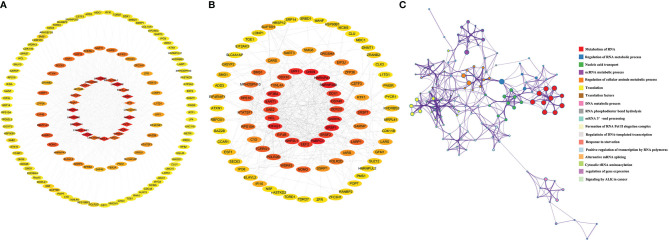
The interaction network of targets protein and gene function prediction of lncRNAs pathways involved in radiosensitivity. **(A)** The interaction network of targets protein of lncRNAs from STRING database (https://www.string-db.org/). **(B)** Coexpression scores based on RNA expression patterns, and on protein co-regulation provided by STRING. **(C)** Gene network of enriched terms by Metascape (https://metascape.org/), a subset of enriched terms has been selected and rendered as a network plot, where terms with a similarity > 0.3 are connected by edges. We select the terms with the best p-values from each of the 20 clusters, with the constraint that there are no more than 15 terms per cluster and no more than 250 terms in total, where each node represents an enriched term.

Then, we searched the target protein of these lncRNAs in RDPDB database (http://rbpdb.ccbr.utoronto.ca/index.php), and conducted the protein-protein interaction (PPI) network based on the STRING database (https://string-db.org/), which covered almost all functional interactions between the expressed proteins (59). Shown as **Figure 2A**, the 24 target proteins were uploaded into the STRING database for analysis. Eighteen nodes and 69 edges were contained in the network; in total, the average node degree is 7.67. Nodes represented the core targets and the extended targets, edges represented the connection between the genes, and the degree value represented the association intensity. The ELAVL1, SRSF1, PTBP1, and HNRNPA1 were the core protein in this PPI network based on degree ranking which was calculated by cytohubba (60). In addition, there was a co-expression relationship between ELAVL1, PTBP1, HNRNPA1, and SRSF proteins, shown as **Figure 2B**. These target genes involved in RNA splicing, mRNA processing, negative regulation of mRNA metabolic process, cellular response to fibroblast growth factors stimulus, and so on, which is shown in **Figure 2C** curved based on Metascape database (http://metascape.org/). Our results indicate that these lncRNAs play key roles in radioresistance during cancer therapy by involving these pathways.

### Meta-Analysis

**Figure 3:**
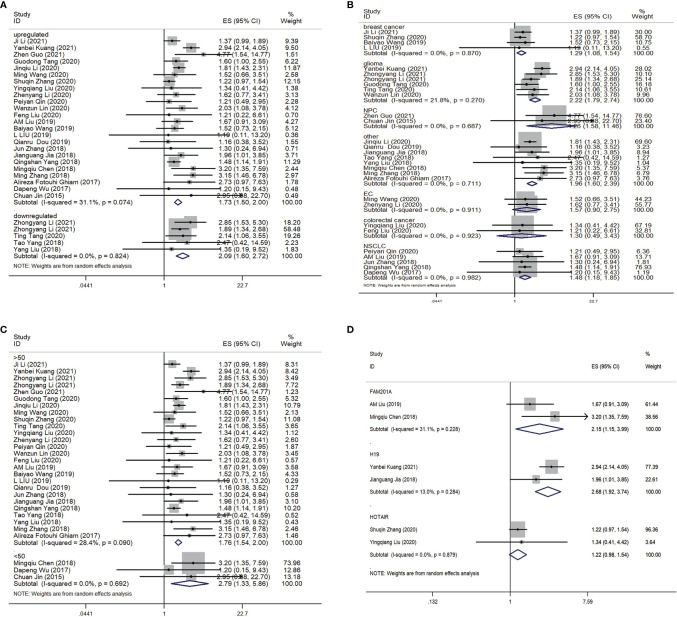
Forest plot for the association between the lncRNAs expression levels with OS. **(A)** Subgroup analysis of the association between lncRNAs expression level and cancer patients according to the up-regulation and down-regelation of lncRNAs. **(B)** Subgroup analysis of the association between lncRNAs expression level and cancer patients according to the difference of cancer types. **(C)** Subgroup analysis of the association between lncRNAs expression level and cancer patients according to the difference of samples size. **(D)** Subgroup analysis of the association between different lncRNAs expression level and the OS of cancer patients. OS, overall survival.

Twenty-nine comparisons were included to evaluate the lncRNAs level response to radioresistance during cancer therapy in cancer patients; enrolling a total of 4986 subjects. Shown as **Figure 3A**, the pooled HR and 95% CIs for the combined up-regulated lncRNAs was 1.73 (95% CI=1.50-2.00; P< 0.01) under the random-effects model, and the pooled HR and 95% CIs for the combined down-regulated lncRNAs was 2.09 (95% CI =1.60-2.72; P<0.01). In addition, subgroup meta-analyses that focused on lncRNAs and the difference of cancers was performed, shown as **Figure 3B**. We found that the dysregulation of lncRNAs was significantly related to poor overall survival (OS) in glioma patients (HR= 2.22, 95% CI =1.79-2.74; P<0.01). Four studies investigated the association between lncRNAs expression and OS in patients with breast cancer, and results indicated that the up-regulated expression of lncRNAs could predict poor OS in patients with breast cancer (HR= 1.29, 95% CI =1.08-1.54; P<0.01). Five studies discussed the relationship between lncRNAs expression with OS in patients with NSCLC, which demonstrated that a higher expression of these five lncRNAs was related to poor OS in NSCLC patients (HR= 1.48, 95% CI =1.18-1.85; P<0.01). Two studies involved the relationship between lncRNAs expression with OS in EC patients. However, the results suggested that a higher expression of these two lncRNAs was not related to OS in EC patients (HR= 1.57, 95% CI =0.90-2.75; P=1.09) and colorectal cancer patients (HR= 1.30, 95% CI =0.49-3.43; P=0.602). Furthermore, subgroup meta-analyses focusing on the sample size was also performed. As shown in **Figure 3C**, the dysregulation of lncRNAs was significantly related to OS in cancer patients no matter if the case sample size was >50 or not. Shown as **Figure 3D**, two studies discussed the relationship between lncRNAFAM201A and the OS in cancer patients (HR=2.15; 95% CI= 1.15-3.99; P <0.01). Another two studies discussed the relationship between lncRNAH19 and the OS in cancer patients (HR=2.68; 95% CI= 1.92-3.74; P <0.01). However, there was no significant relationship between lncRNA HOTAIR and the OS in cancer patients (HR=1.22; 95% CI= 0.98-1.54; P =0.08).

In the present meta-analysis, sensitivity analysis and publication bias were also performed. Egger’s test and Begg’s showed that there was no risk of publication bias in this meta-analysis, shown as **Supplementary Figure S1**.

## Discussion

There is a growing investigation to demonstrate that lncRNAs play an important role in radioresistance or radiosensitivity. However, previous individual studies couldn’t provide strong evidence due to small sample size and different research standards. Therefore, we performed this present meta-analysis to evaluate the relationship between the expression level of lncRNAs and radioresistance or radiosensitivity in cancer patients. Here, we retrieved all publicly available lncRNAs and integrated data extracted from 19 related articles including 4986 cancer subjects. The results indicate that the upregulation or downregulation of lncRNAs are significantly related to radioresistance response in cancer patients. Down-regulation of TPTEP1, MEG3, and UCA1 and up-regulation of LINC01057, LINC00520, CCAT2, HOTAIR, Rpph1, FAM201A, LINC02582, LINC00511, FAM83H-AS1, H19, CYTOR, LINC00483, NKILA, PVT1, and MALAT1 are deeply involved in the radiotherapy of cancer patients. These findings are novel and provide insights into the potential biological markers in targeting radiotherapy response.

Traditionally, there are four kinds of biomarkers in cancer biology, including diagnostic biomarkers, prognostic biomarkers, diagnostic companion biomarkers, and predictive theragnostic biomarkers. The last biomarker had been used as an indicator of normal biological processes, pathogenic processes, or responses to an exposure or intervention (61). Consequently, exploring novel uses of each type of biomarker, especially predictive theragnostic biomarkers, is crucial to improving patient management and treatment outcome. However, radioresistance is a major factor that leads to poor prognosis for cancer patients. Radiotherapy always induced DNA damage or excessive ROS by ionizing radiation to activate apoptotic signaling pathways in cancer cells, leading to cell death (62). In addition, crucial molecules at cell cycle checkpoints could regulate and arrest cell cycle progression. Therefore, cell cycle arrest was closely related to radioresistance or radiosensitivity response. For example, down-regulation of metastasis-associated lung adenocarcinoma transcript 1 (MALAT1) could negatively regulate miR-145 levels and increase radiosensitivity, which may induce G2/M arrest and affect DNA repair (56). However, MALAT1 could also induce the radioresistance response during esophageal squamous cell carcinoma (ESCC) treatment. The reason was that MALAT1 decreased the expression of CDC kinase subunit 1 (CKS1) and increased the G2/M arrest response by inhibiting the expression of p27 (63). Similarly, the hox transcript antisense intergenic lncRNA (HOTAIR) increased radioresistance response by decreasing the level of p21 and inducing cell cycle arrest at the S phase (64). Furthermore, our meta-analysis revealed that overexpression of MALAT1 and HOTAIR contributes to increased radioresistance in NPC, colorectal cancer, and breast cancer patients. Except for DNA damage, apoptosis was the main mode of cancer cell death induced by radiotherapy (65). For example, HOTAIR acted as a key inhibitor to partly turn off the Wnt/β-catenin pathway in pancreatic ductal adenocarcinoma cells (66), and as a result, the radiosensitivity response was reduced. Increasing the level of urothelial carcinoma associated 1 (UCA1) induced the radioresistance response by increasing Akt activation in prostate cancer cells (54), which was consistent with the result from our meta-analysis. However, lncRNAs modulated gene expression through multiple mechanisms. For example, they could act as competing endogenous RNAs (ceRNAs) to prevent miRNA from interacting with shared mRNA, resulting a lncRNA-miRNA-mRNA regulatory network to affect the expression of downstream target (43). For example, lncRNA colon-cancer-associated transcript-1 (CCAT1) negatively regulated miR-148b expression (23) and 201-member A (FAM201A) (45) regulated ataxia telangiectasia mutation and mammalian target of rapamycin (mTOR) expression *via* miR-101 (26)to mediate the radiosensitivity of breast cancer and ESCC. In addition, the lncRNAs plasmacytoma variant translocation 1 (PVT1) acted as sponger of miR-195 to reduce radioresistance of NSCLC (55). Therefore, combined with our meta-analysis, we could draw a conclusion that all of these lncRNAs could act as potential predictive markers for radioresistance response.

In the present meta-analysis, tissue samples were used to assess lncRNAs expression in the included literature. However, lncRNAs from ncRNAs from serum or plasma samples allow for more collection, lower expense, and more convenient analysis. Therefore, there are several limitations in this meta-analysis. First, there were a small number of eligible studies, and some of the sample sizes included in these studies were < 50. Second, these eligible studies explored multiple kinds of cancers or tumors, so there were not enough studies to calculate the pooled HR value of one sole kind of cancer. Third, most studies were conducted in China and only one study was investigated in Caucasians. Therefore, the results may not interpret well to populations all over the world. Finally, most studies explored absolute differences in lncRNAs and HR values were applied for the current meta-analysis, which may lead to bias and decreased accuracy of the results. Further studies are necessary to be conducted with larger samples, multiple centers, populations, as well as more kinds of cancer.

## Conclusion

In conclusion, this systematic review and meta-analysis explored the association between lncRNAs expression and radiotherapeutic response in various cancers. Furthermore, our meta-analysis provided a series of potential lncRNAs which could indicate patients at high risk of radresistance response; allowing doctors to improve their therapeutic strategies to overcome radresistance. Therefore, more work is needed to explore how lncRNAs interact with signaling molecules to induce radiosensitivity or radioresistance as well as to evaluate the therapeutic diagnosis value of lncRNAs in radiotherapy.

## Data Availability Statement

The raw data supporting the conclusions of this article will be available by email the corresponding authors.

## Author Contributions

PL and GM were responsible for the design of this work. WX and QR performed the database search and data collection. WX and QW was responsible for the data analysis. JY and PL drafted this article. All authors reviewed this draft and approved the final manuscript.

## Funding

This study was supported by the project of Science and Technology plan of Hangzhou Municipal Health Bureau in 2018 (2018Z05); the projection of Zhejiang Health Commission (2018KY593); the project of Hangzhou Medical Key Discipline Construction; the Natural Science Foundation of Zhejiang Province (LQ20H250001), funds from the Health Bureau of Zhejiang Province (2019RC091, 2020ZB009), the project of Hangzhou technology plan (ZD20220005).

## Conflict of Interest

The authors declare that the research was conducted in the absence of any commercial or financial relationships that could be construed as a potential conflict of interest.

## Publisher’s Note

All claims expressed in this article are solely those of the authors and do not necessarily represent those of their affiliated organizations, or those of the publisher, the editors and the reviewers. Any product that may be evaluated in this article, or claim that may be made by its manufacturer, is not guaranteed or endorsed by the publisher.
