# Supplementary material for: LncRNAs as Theragnostic Biomarkers for Predicting Radioresistance in Cancer: A Systematic Review and Meta-Analysis
Source: Front Oncol. 2022 May 25;12:767750. doi: 10.3389/fonc.2022.767750 (PMC9176206; doi:10.3389/fonc.2022.767750)
Supplement: Supplementary file 1 [file DataSheet_1.docx]

Supplementary Material

# Supplementary Figures


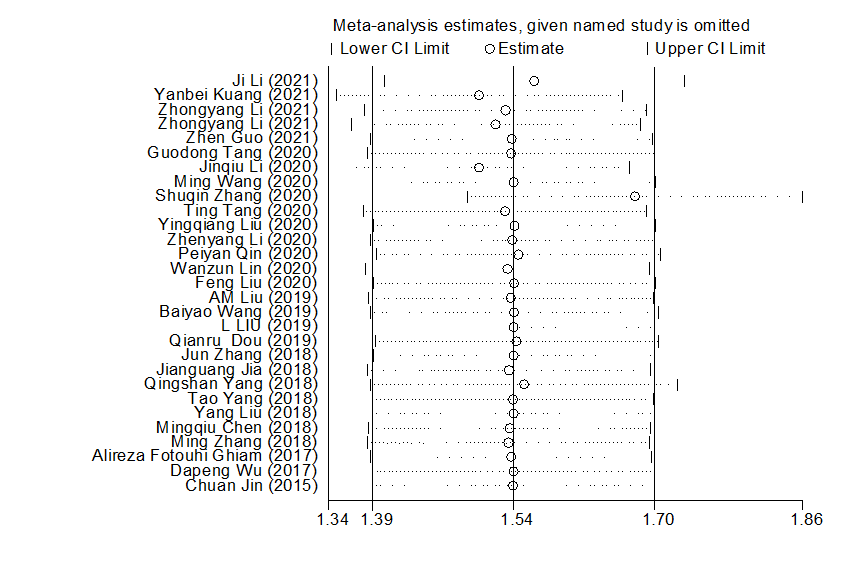


# Supplementary Figure 1. The sensitivity analysis of the included articles.

#

# Supplementary Figure 2. The publication bias assessment of the included articles.

# Supplementary Tables

| **Supplementary Table 2. Details of study quality evaluation via the Newcastle-Ottawa Scale.** | | | | | | | | | | |
| --- | --- | --- | --- | --- | --- | --- | --- | --- | --- | --- |
| **Study** | **Representativeness**  **of the exposed**  **cohort** | **Selection of the non-exposed**  **cohort** | **Ascertainment**  **of exposure** | **Outcome**  **not present**  **at baseline** | **Control for age**  **and gender** | **Control for other con-founding**  **factors** | **Assessment**  **of outcome** | **Enough long follow-up duration** | **Adequacy of follow-up of cohorts** | **Total** |
| **Ji Li** | **1** | **1** | **1** | **0** | **0** | **0** | **1** | **1** | **1** | **6** |
| **Yanbei Kuang** | **0** | **1** | **1** | **1** | **0** | **0** | **0** | **1** | **1** | **5** |
| **Zhongyang Li** | **1** | **1** | **1** | **0** | **0** | **0** | **1** | **1** | **1** | **6** |
| **Zhen Guo** | **1** | **1** | **1** | **1** | **0** | **0** | **1** | **1** | **1** | **7** |
| **Wanzun Lin** | **1** | **1** | **0** | **0** | **0** | **0** | **1** | **1** | **1** | **5** |
| **Feng Liu** | **1** | **1** | **1** | **0** | **0** | **0** | **1** | **1** | **1** | **6** |
| **Peiyan Qin** | **1** | **1** | **1** | **0** | **0** | **0** | **1** | **1** | **1** | **6** |
| **Guodong Tang** | **1** | **1** | **1** | **0** | **1** | **0** | **1** | **1** | **1** | **7** |
| **Jinqiu Li** | **1** | **1** | **1** | **0** | **0** | **0** | **1** | **1** | **1** | **6** |
| **Ming Wang** | **1** | **1** | **1** | **0** | **1** | **0** | **1** | **1** | **1** | **7** |
| **Shuqin Zhang** | **1** | **1** | **1** | **1** | **1** | **1** | **1** | **1** | **1** | **9** |
| **Ting Tang** | **1** | **1** | **1** | **0** | **1** | **0** | **1** | **1** | **1** | **7** |
| **Yingqiang Liu** | **0** | **1** | **0** | **1** | **0** | **1** | **1** | **1** | **1** | **6** |
| **Zhenyang Li** | **1** | **0** | **1** | **0** | **1** | **0** | **1** | **1** | **1** | **6** |
| **AM Liu** | **1** | **1** | **1** | **1** | **0** | **1** | **1** | **1** | **1** | **8** |
| **Baiyao Wang** | **1** | **1** | **1** | **1** | **0** | **0** | **1** | **1** | **1** | **7** |
| **L LIU** | **1** | **1** | **1** | **0** | **0** | **1** | **1** | **1** | **1** | **7** |
| **Qianru Dou** | **1** | **1** | **1** | **1** | **1** | **0** | **0** | **1** | **1** | **7** |
| **Jianguang Jia** | **1** | **1** | **1** | **0** | **0** | **1** | **1** | **1** | **1** | **7** |
| **Jun Zhang** | **1** | **1** | **1** | **0** | **0** | **0** | **1** | **1** | **1** | **6** |
| **Qingshan Yang** | **1** | **1** | **1** | **1** | **1** | **0** | **1** | **1** | **1** | **8** |
| **Tao Yang** | **1** | **1** | **1** | **0** | **0** | **0** | **1** | **1** | **1** | **6** |
| **Yang Liu** | **1** | **0** | **1** | **0** | **0** | **1** | **1** | **1** | **1** | **6** |
| **Mingqiu Chen** | **1** | **1** | **1** | **0** | **0** | **0** | **1** | **1** | **1** | **6** |
| **Ming Zhang** | **1** | **1** | **1** | **0** | **0** | **1** | **1** | **1** | **1** | **7** |
| **Alireza F Ghiam** | **1** | **1** | **1** | **1** | **1** | **0** | **1** | **1** | **1** | **8** |
| **Dapeng Wu** | **1** | **1** | **0** | **1** | **1** | **0** | **1** | **1** | **1** | **8** |
| **Chuan Jin** | **1** | **1** | **1** | **0** | **0** | **0** | **1** | **1** | **1** | **6** |
